# Decreased Levels of Bisecting GlcNAc Glycoforms of IgG Are Associated with Human Longevity

**DOI:** 10.1371/journal.pone.0012566

**Published:** 2010-09-07

**Authors:** L. Renee Ruhaak, Hae-Won Uh, Marian Beekman, Carolien A. M. Koeleman, Cornelis H. Hokke, Rudi G. J. Westendorp, Manfred Wuhrer, Jeanine J. Houwing-Duistermaat, P. Eline Slagboom, André M. Deelder

**Affiliations:** 1 Biomolecular Mass Spectrometry Unit, Department of Parasitology, Leiden University Medical Center, Leiden, The Netherlands; 2 Section Medical Statistics, Department of Medical Statistics and Bioinformatics, Leiden University Medical Center, Leiden, The Netherlands; 3 Section Molecular Epidemiology, Department of Medical Statistics and Bioinformatics, Leiden University Medical Center, Leiden, The Netherlands; 4 Department of Gerontology and Geriatrics, Leiden University Medical Center, Leiden, The Netherlands; Universidad Europea de Madrid, Spain

## Abstract

**Background:**

Markers for longevity that reflect the health condition and predict healthy aging are extremely scarce. Such markers are, however, valuable in aging research. It has been shown previously that the N-glycosylation pattern of human immunoglobulin G (IgG) is age-dependent. Here we investigate whether N-linked glycans reflect early features of human longevity.

**Methodology/Principal Findings:**

The Leiden Longevity Study (LLS) consists of nonagenarian sibling pairs, their offspring, and partners of the offspring serving as control. IgG subclass specific glycosylation patterns were obtained from 1967 participants in the LLS by MALDI-TOF-MS analysis of tryptic IgG Fc glycopeptides. Several regression strategies were applied to evaluate the association of IgG glycosylation with age, sex, and longevity. The degree of galactosylation of IgG decreased with increasing age. For the galactosylated glycoforms the incidence of bisecting GlcNAc increased as a function of age. Sex-related differences were observed at ages below 60 years. Compared to males, younger females had higher galactosylation, which decreased stronger with increasing age, resulting in similar galactosylation for both sexes from 60 onwards. In younger participants (<60 years of age), but not in the older age group (>60 years), decreased levels of non-galactosylated glycoforms containing a bisecting GlcNAc reflected early features of longevity.

**Conclusions/Significance:**

We here describe IgG glycoforms associated with calendar age at all ages and the propensity for longevity before middle age. As modulation of IgG effector functions has been described for various IgG glycosylation features, a modulatory effect may be expected for the longevity marker described in this study.

## Introduction

Human aging research would be greatly facilitated if markers were available that reflect the physiological state of the human body and predict morbidity and mortality. Such markers indicate biological age of individuals instead of calendar age, but have so far hardly been identified [1]. Markers of calendar age have been described frequently [2].

Among the classes of biomolecules that might reflect mechanisms of biological aging are the sugar chains on proteins and lipids, which are called glycans. All cells as well as most secreted proteins carry a set of glycans. These glycans, generated by enzymatic reactions (not to be confused with non-enzymatic glycation), play important roles, e.g. in cell-cell interactions, cell-matrix interactions, molecular trafficking, receptor activation, and other biological and immunological events [3]. Several classes of glycans exist, among which the proteoglycans, glycosphingolipid glycans, O-glycans and N-glycans. In this study we focus on the N-glycans, which are sugar chains covalently attached to asparagine residues of proteins. N-glycans all have a common core-structure, consisting of an N-acetylglucosamine (GlcNAc) attached to the asparagine, to which a second GlcNAc and three mannoses are attached. This core may carry a multitude of different glycan motifs. The biosynthesis of N-glycans is not regulated by a template, as is the case with proteins, but is mainly dependent on the expression and activity of specific glycosyltransferases in a cell. Therefore, a glycoprotein normally exists as a heterogeneous population of glycoforms which carry different glycans on the same protein backbone or even the same glycosylation site. Moreover, shifts in protein glycosylation patterns reflect regulated modulations of the glycosylation machinery of the different cells producing that particular glycoprotein.

The most common type of N-glycans of plasma proteins is the complex type. In the biosynthetic route to this N-glycan type, several GlcNAc transferases attach GlcNAc residues to the mannoses of the glycan core, which can be further extended by galactose, sialic acid and fucose residues.

Differences in N-glycosylation patterns of plasma proteins have been associated with several diseases including rheumatoid arthritis, malignancies, liver diseases and diabetes [4]–[7], and it may be hypothesized that one or more aspects of glycosylation reflect the overall health status, and could as such constitute markers for biological age.

Associations of total plasma protein glycosylation patterns with calendar age have recently been evaluated in a study population of 100 Belgian individuals, subdivided in five sex-matched groups of 20, 30, 40, 50 and 60 years of age [8]. As compared to subjects of 20 years of age, elderly individuals of ages above 50 had increased levels of non-galactosylated glycans, while the levels of galactosylated structures decreased with increasing calendar age. In the same study, a population of 120 Italian centenarians was compared to 79 elderly (mean age 81) and 63 middle-aged (mean age 44) individuals. In this high-age population, changes in plasma protein glycosylation were observed as a function of age, which were similar to those observed for the Belgian population. This indicates that the changes in plasma protein glycosylation with age can be extrapolated to very high ages. Since long-lived subjects are considered to show delayed aging, it would be expected that centenarians reveal glycosylation patterns comparable to younger age groups; however this was not the case.

A number of studies have also provided evidence that among the plasma proteins, specifically the glycosylation of immunoglobulins (IgGs) changes with human aging. Parekh *et al*. reported in 1988 decreased galactosylation of IgG as a function of increasing age [9]. In subsequent studies the rate of the age-dependent changes in galactosylation levels of IgG were shown to be sex-specific [10], [11], while also increased levels of glycans with bisecting GlcNAc were reported to associate with higher age [10], [11]. These results were corroborated in the study with Italian centenarians [8]. Although the studies mentioned above provided valuable information regarding changes in glycosylation with respect to calendar age, the question whether they might reflect biological age remains open.

Studies into markers for biological age require a specific study design: nonagenarian sibling pairs, their offspring and partners of the offspring were recruited in the Leiden Longevity Study (LLS) [12]. Longevity is a familial trait in these subjects since a 30% survival advantage over the general population was observed for the parents of the sibling pairs, the brothers and sisters, and the offspring. It was recently reported [1] that large LDL cholesterol particle size is an early hallmark of longevity in this cohort, present both in the sibling pairs as well as their offspring when compared to the partners of the offspring as controls.

Here we address the question whether glycosylation of plasma IgG could likewise be an early hallmark of human longevity. To this end, we investigated whether previous findings regarding the relation between galactosylation and calendar age could be confirmed in participants of the LLS, and subsequently evaluated whether IgG glycosylation is associated with human familial longevity.

## Results

**Figure 1:**
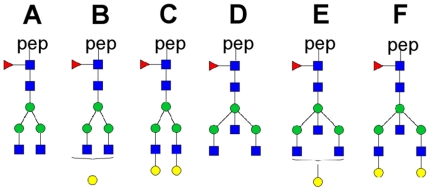
Structures of the N-glycans attached to IgG glycopeptides, as profiled in our analysis. Compositions and structural schemes are given in terms of *N*-acetylglucosamine (blue square), mannose (green circle), galactose (yellow circle) and fucose (red triangle).

**Figure 2:**
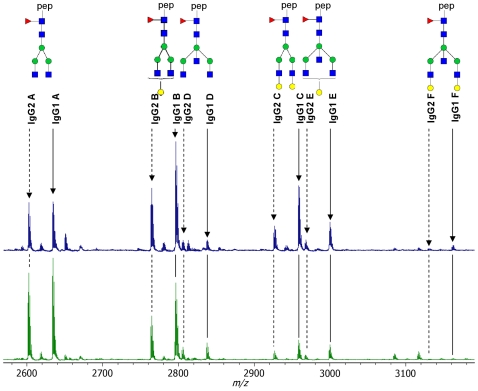
MALDI-TOF-MS spectra of representatives of a young and an old participant. MALDI-TOF-MS spectra were acquired in the reflectron-positive mode with α-cyano-4-hydroxy-cinnamic acid matrix spotted on top of the sample. Compositions and structural schemes as in Figure 1.

IgG was isolated from plasma samples of a subset of 1967 participants (1287 offspring of long-lived siblings and 680 partners of the offspring) of the Leiden Longevity Study (Table 1). Fc glycosylation patterns of IgG1 and IgG2 were investigated by analysis of tryptic glycopeptides using MALDI-TOF mass spectrometry. Six glycoforms per IgG subclass were determined by MALDI-TOF-MS (Figure 1 and 2). Since the intensities of all glycoforms were related to the monogalactosylated, core-fucosylated biantennary species (glycoform **B**), five relative intensities were registered per IgG subclass.

**Table 1:** Numbers of individuals and average ages of the participants.

*Variable*	*Total*	*Offspring*			*Partners of offspring*		
		*Total*	*Male*	*Female*	*Total*	*Male*	*Female*
**Nr. of individuals**	1967	1287	635	652	680	289	391
**Mean age in Years (range)**	59.22 (30.2–79.2)	59.32(33.6–77.6)	59.50 (33.6–77.6)	59.14 (42.0–76.3)	59.04 (30.2–79.2)	61.53 (30.2–79.2)	57.20 (30.2–74.9)

The mean age, stratified per sex, is depicted.

### Associations of IgG glycosylation with calendar age and sex, and the interaction between sex and calendar age

A large age-range was covered by offspring and partners in the Leiden Longevity Study (age 30.2–79.2 years), hence we examined the association of IgG glycosylation with age by linear regression analysis. The results are shown in Table 2. Clearly, galactosylation tends to decrease with calendar age as di-galactosylated glycopeptides (**C** and **F**) are less abundant in older participants, while non-galactosylated glycopeptides (**A** and **D**) are more abundant in older participants. This is illustrated in Figure 2, where a typical ‘young’ (with low **A, D**, and high **C, F**) and a typical ‘old’ (with high **A, D**, and low **C, F**) MALDI-TOF mass spectrum are presented.

**Table 2:** Regression coefficients from the linear regression analysis for the response variable, IgG values, and the covariates age and sex (female  = 0, male  = 1) after adjustment for the family status.

*IgG subclass and glycoform*	*Age*		*Sex*	
	*change per year of calendar age (SE)*	*P*	*difference between female and male (SE)*	*P*
IgG1 A	0.017 (0.001)	0.000		
IgG1 C	−0.014 (0.001)	0.000		
IgG1 D	0.019 (0.001)	0.000	−0.033 (0.015)	0.035
IgG1 E			−0.036 (0.011)	0.001
IgG1 F	−0.009 (0.001)	0.000		
IgG2 A	0.019 (0.001)	0.000	−0.024 (0.011)	0.035
IgG2 C	−0.015 (0.001)	0.000	0.025 (0.010)	0.007
IgG2 D	0.019 (0.001)	0.000	−0.081 (0.015)	0.000
IgG2 E			−0.051 (0.012)	0.000
IgG2 F	−0.006 (0.001)	0.000		
IgG1 D/A			−0.037 (0.011)	0.001
IgG1 F/C	0.005 (0.001)	0.000		
IgG2 D/A			−0.058 (0.011)	0.000
IgG2 F/C	0.009 (0.001)	0.000	−0.051 (0.017)	0.003

Only significant results (*P<0.05*) are depicted. Positive regression coefficients for age indicate increased levels with increasing age, while negative coefficients indicate decreased levels with increasing age. For sex, a positive regression coefficient indicates higher levels for male participants than female participants, while a negative coefficient indicates lower levels for male participants.

Previous publications have shown that sex-specific differences in IgG glycosylation exist and that the relation between age and IgG glycosylation is sex-dependent [8], [10], [11]. Significant differences in IgG glycosylation were indeed observed between the sexes. For both IgG1 and IgG2 the glycoforms **D** and **E** were more prevalent in females than in males, while the **A** and **C** glycoforms were respectively more and less prevalent in females, but only for the IgG2 subclass (Table 2).

**Figure 3:**
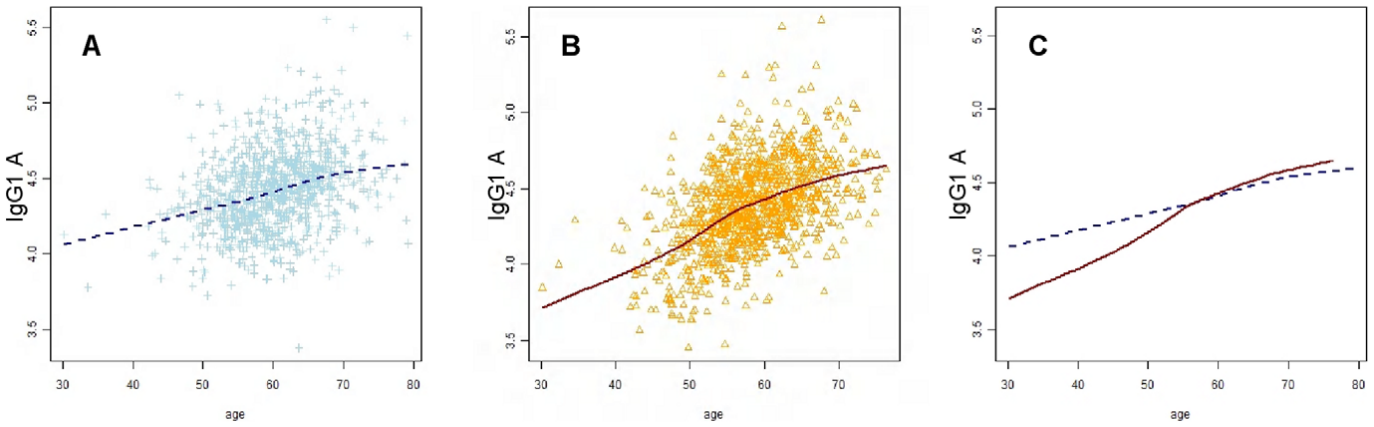
IgG glycosylation and the interaction between age and sex. Relationship between age and IgG1 **A** glycoform, stratified for sex. Males are plotted in blue, with a fitted line in dashed dark blue (A), while females are plotted in orange with a fitted line in continuous dark red (B). Both lines were fitted using the loess (locally weighted scatter plot smoothing) method, and plotted for both males in females (C) to illustrate the crossing slopes.

Subsequently, we evaluated whether the calendar age-dependent decrease in galactosylation is sex-specific. Highly significant interactions between age and sex were observed for all IgG glycoforms, which is illustrated by the crossing slopes of the males and females for IgG1 **A** in Figure 3. Up to 60 years of age, females tend to have stronger associations between IgG glycosylation and age than males. From that time point onwards, IgG glycosylation changes with age are similar for females and males.

To evaluate a possible association of calendar age with a so-called bisecting GlcNAc, the GlcNAc which is attached to the β-linked mannose, we calculated the ratio bisected/non-bisected for both di-galactosylated and non-galactosylated glycoforms, **F**/**C** and **D**/**A** respectively. The resulting regression coefficients are depicted in Table 2, and the ratios for galactosylated compounds tend to increase with increasing age. In other words, the relative incidence of structures with bisecting GlcNAc among the galactosylated species is increasing with age. This is in accordance with findings by Yamada *et al.* and Shikata *et al.* [10], [11]. No interaction between sex and age was observed for the non-bisecting/bisecting ratio's, indicating that the interaction for the non-bisecting glycoform is similar to the interaction for the bisecting glycoform.

### Associations of IgG glycosylation and longevity

The Leiden Longevity Study design allows the testing for association between glycosylation and familial longevity by comparing the data of offspring and partners. Logistic regression analysis performed on the complete dataset did not show significant differences between these groups. Since the relation between IgG glycosylation and age differed for females and males at ages below 60 years (Figure 3), we analyzed the data stratified for ages below and above 60 years applying logistic regression analysis. Three glycopeptides, all with bisecting GlcNAc and low galactosylation, were shown to be lowered in offspring compared to their partners at younger age, but not at older age, as depicted in Table 3. Upon further stratification for sex, the associations between family status and IgG glycosylation were lost, as the groups were too small.

**Table 3:** Odds ratios and p-values from logistic regression.

*IgG subclass and glycoform*	*Age<60 (n0 = 372, n1 = 701)*		*Age> = 60 (n0 = 308, n1 = 586)*	
	*Odds ratio (95% C.I.)*	*P*	*Odds ratio (95% C.I.)*	*P*
**IgG1 A**	0.75 (0.43–1.30)	0.302	0.99 (0.55–1.75)	0.962
**IgG1 C**	0.85 (0.46–1.58)	0.613	0.91 (0.46–1.80)	0.792
**IgG1 D**	**0.52 (0.34–0.80)**	**0.003**	1.04 (0.66–1.64)	0.882
**IgG1 E**	**0.47 (0.26–0.84)**	**0.011**	1.13 (0.63–2.01)	0.679
**IgG1 F**	0.68 (0.45–1.03)	0.071	1.03 (0.66–1.61)	0.901
**IgG2 A**	0.79 (0.45–1.40)	0.425	1.22 (0.68–2.16)	0.506
**IgG2 C**	0.92 (0.49–1.72)	0.798	0.75 (0.37–1.52)	0.426
**IgG2 D**	**0.53 (0.33–0.83)**	**0.006**	1.10 (0.72–1.70)	0.659
**IgG2 E**	0.58 (0.32–1.05)	0.071	1.07 (0.64–1.79)	0.806
**IgG2 F**	0.73 (0.51–1.04)	0.085	1.04 (0.75–1.44)	0.810

Data were split in two groups: one with individuals below 60 years of age, and one with individuals above 60 years of age. 0 =  partners, 1 =  offspring. When Odds Ratio (OR) is reported lower than 1, it indicates that subjects with lower IgG glycosylation values tend to belong to the group of offspring.

**Figure 4:**
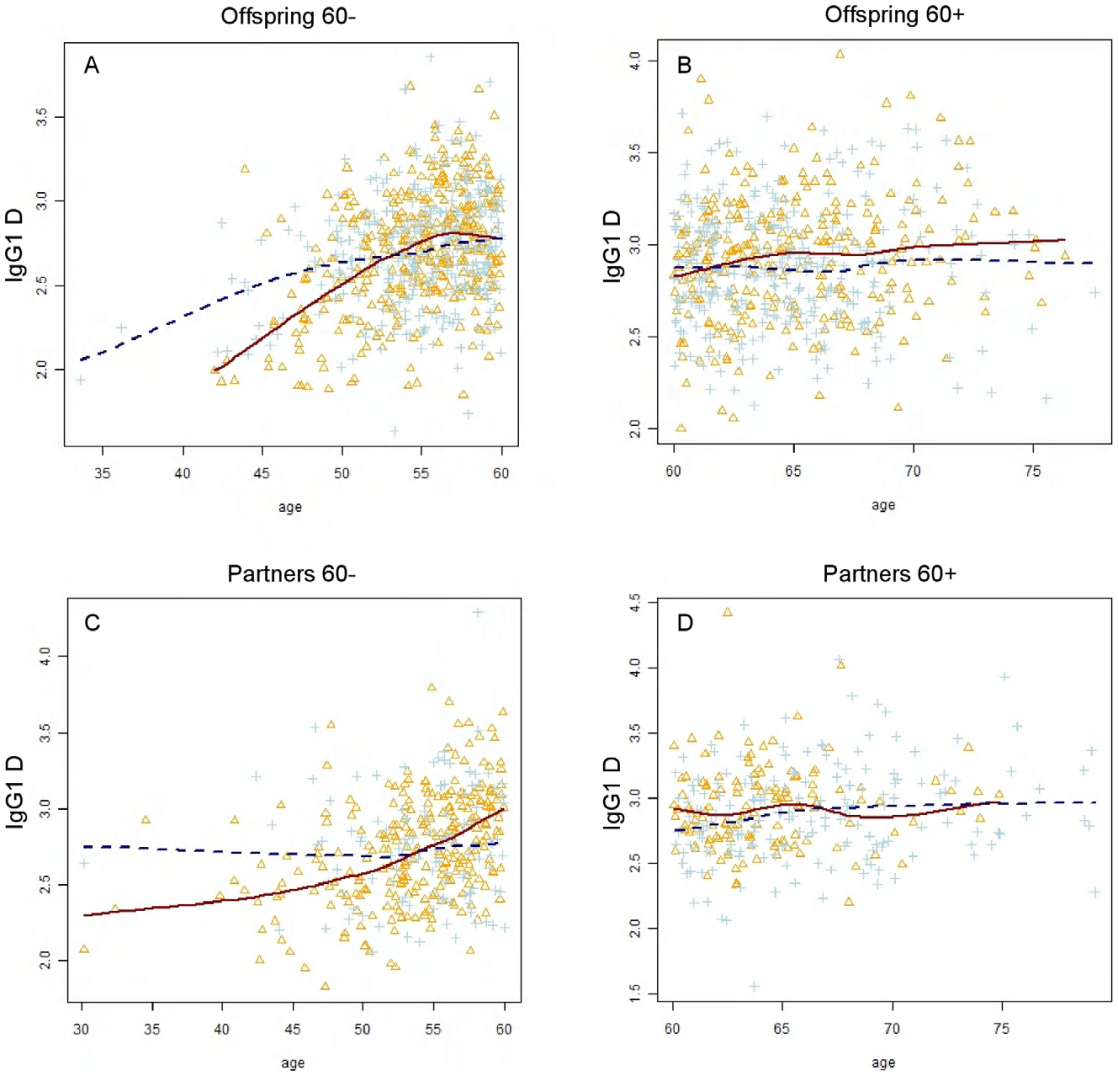
IgG glycosylation and its association to longevity. Relationship between age and IgG1 **D** glycoform, stratified for the family status and younger/older than 60 years. Values from males are plotted in blue, while values from females are plotted in orange. The fitted lines (dashed dark blue for males and continuous dark red for females) are drawn using the loess (locally weighted scatter plot smoothing) method.

To further elaborate on the role of structures carrying a bisecting GlcNAc in familial longevity, logistic regression analysis was performed on the bisecting/non-bisecting ratios for the stratified age groups. The results are shown in Table 4. At ages below 60, the offspring tends to have a lower amount of bisecting GlcNAc compared to their partners, but only for non-galactosylated glycoforms. This holds for both IgG1 and IgG2. However, as previously found (Table 3), no differences could be observed above 60 years of age, thus confirming the association of the presence of a bisecting GlcNAc on non-galactosylated N-glycans from IgG with familial longevity at younger ages. Since the bisected/non-bisected ratio for the mono-galactosylated glycans is already determined in glycoform **E** due to the normalization on the mono-galactosylated non-bisected glycoform **B**, our results indicate that the presence of a bisecting GlcNAc on non- (Table 3 and 4) and mono-galactosylated (Table 3) IgG1 as well as non-galactosylated (Table 3 and 4) IgG2 N-glycans is an early marker for familial longevity. To illustrate this, the relation between age and IgG glycosylation is plotted for IgG1 **D**, stratified for family status, age group and gender (Figure 4). Clearly, the relative amount of IgG1 **D** is lowered in the offspring compared to their partners, but only below 60 years of age.

**Table 4:** Logistic regression analysis of non-bisecting/bisecting ratios with the response variable family status.

*IgG subclass and ratio*	*Age<60 (n0 = 372, n1 = 701)*		*Age> = 60 (n0 = 308, n1 = 586)*	
	*Odds ratio (95% C.I.)*	*p-value*	*Odds ratio (95% C.I.)*	*p-value*
**IgG1 D/A**	**0.41 (0.23–0.74)**	**0.003**	1.09 (0.58–2.03)	0.796
**IgG1 E^1^**	**0.47 (0.26–0.84)**	**0.011**	1.13 (0.63–2.01)	0.679
**IgG1 F/C**	0.62 (0.37–1.04)	0.069	1.10 (0.63–1.90)	0.727
**IgG2 D/A**	**0.43 (0.24–0.78)**	**0.006**	0.98 (0.56–1.72)	0.941
**IgG2 E^1^**	0.58 (0.32–1.05)	0.071	1.07 (0.64–1.79)	0.806
**IgG2 F/C**	0.69 (0.45–1.05)	0.083	1.13 (0.79–1.62)	0.508

Age groups are stratified <60 and >60. * the value for glycoform **E** is already a bisecting/non-bisecting ratio as it is normalized on its non-bisected counterform **B**.

To evaluate the predictive ability of the association between IgG glycosylation and longevity, we investigated the areas under the ROC curves (AUC) of the IgG1 **D**, IgG1 **E** and IgG2 **D** glycoforms. Only subjects younger than 60 years of age were included. For all three AUC's values of around 0.6 were obtained, indicating that multiple markers are needed for the prediction of complex phenotypes, like familial longevity.

As IgG glycosylation has previously been shown to be associated with several disease states (e.g. [4]–[7]) it was assessed whether such associations could interfere with the observed relationship between IgG glycosylation and longevity. As most diseases associated with changes in IgG glycosylation comprise inflammatory components, the association between glycosylation and levels of C-reactive protein, a marker for inflammation, was evaluated. While all non-galactosylated glycoforms were positively associated with levels of CRP (P<0.001 for all glycoforms), di-galactosylated glycopeptides (except from IgG2 **F**) were negatively associated with CRP levels (P<0.002 for all glycoforms). Upon addition of the CRP level in the logistic regression model as a covariate, the association between glycosylation and longevity remained significant (P<0.05), indicating that the association is not affected by individuals carrying an inflammatory disease.

To further corroborate these results, individuals which had been diagnosed with malignancies or diabetes, two major age-related diseases, were excluded from the analysis; this resulted in a reduction of the cohort size of 28%. The associations between IgG glycoforms (IgG1 **D**, IgG1 **E** and IgG2 **D**) and longevity remained significant (P = 0.037, P = 0.034 and P = 0.010, respectively) below 60 years of age. This confirms that the association between IgG glycosylation and familial longevity is not affected by diseased individuals.

## Discussion

This is the first large-scale study in which IgG glycosylation patterns have been evaluated for association with familial longevity and calendar age in one design. Decreased levels of bisecting GlcNAc in the context of non-galactosylated glycans of IgG1 and IgG2 were associated with familial longevity in younger participants (<60 years of age, see Table 3, glycoforms **D**). This glycoform was also affected by calendar age (see Table 2, glycoform **D**) and since the relative levels of non-galactosylated glycans containing a bisecting GlcNAc increase with calendar age, while the offspring is associated with lowered values, the offspring has a ‘younger’ profile. The offspring was previously found to be more healthy than their partners [13], therefore it may be speculated that the non-galactosylated glycan containing a bisecting GlcNAc is reflecting younger biological age or better health condition of the offspring as compared to their partners.

In the case of non-galactosylated glycans, the bisecting/non-bisecting ratio does not associate with calendar age, but again a decreased ratio for non-galactosylated glycoforms is associated with the offspring (Table 4, **D**/**A**).

In this study, comprising 1967 participants in the age range of 30 to 79 years, a clear tendency of decreased galactosylation with increasing calendar age was observed (Table 2). This conclusively confirms earlier data from several other studies on healthy individuals [8]–[11]. In one of these studies, Shikata *et al*. observed decreased galactosylation with increased age only for female individuals [10], while, interestingly, we observed this effect for both sexes. Since female individuals show stronger associations between calendar age and decreased galactosylation than males up to 60 years of age, it might be speculated that the cohort studied by Shikata *et al.*, was only large enough to reveal associations for female individuals, but not for male individuals.

In addition, a tendency towards increasing levels of bisecting GlcNAc was observed with increasing age. This could be observed for both sexes, but only in di-galactosylated glycoforms (Table 2, **F**/**C**). Moreover, we showed for the first time that glycosylation features of IgG subclasses 1 and 2 have comparable associations with calendar age, although sex-specific differences were observed for these subclasses.

As immunoglobulin G is produced in B-lymphocytes, differences in glycosylation patterns of IgG are postulated to be a reflection of altered N-glycan biosynthesis in B-lymphocytes. The decrease in abundance of non-galactosylated glycopeptides carrying a bisecting GlcNAc with longevity presumably reflects a decreased activity of GlcNAc transferase III, the enzyme that catalyzes the addition of a β-1,4-linked GlcNAc to the β-linked mannose of the trimannosyl core [14], and an increase of the β4-galactosyltransferase(s), which catalyzes the addition of β-1,4-linked galactoses to the GlcNAc residues [15], [16]. In an alternative, but less likely explanation the changes could be caused by alterations in production or location of activated sugar nucleotide donors. Only non-galactosylated glycoforms containing a bisecting GlcNAc were shown to be associated with familial longevity, while the bisecting GlcNAc alone as well as the level of galactosylation were not associated. The activities of the glycosyltransferases involved – GlcNAc transferase III and at least one galactosyltransferase – might be negatively correlated, possibly by concerted regulation of transcription of the respective genes.

However, an alternative hypothesis would be that the GlcNAc transferase III tends to be less active, while, independently, the galactosyltransferases are more active in offspring versus partners. All glycopeptides containing a bisecting GlcNAc would then be decreased with familial longevity and all glycopeptides with galactoses would be increased with familial longevity. The changes in glycosylation pattern between the offspring and their partners are small, therefore significant association could only be found for the glycans where both effects are changing the levels of a glycoform in the same direction: the non-galactosylated glycoforms containing a bisecting GlcNAc.

Alterations of the glycosylation of the Fc part of IgGs have been shown to modulate Fc receptor binding. The presence of a bisecting GlcNAc on an anti-neuroblastoma IgG1 was shown to increase antibody-dependent cellular cytotoxicity (ADCC) mediated by binding of the antibody to the Fcγ-receptor [17], while the presence of non-fucosylated glycans on the anti-CD-20 IgG1 rituximab was shown to enhance ADCC through its high binding to both the FcγRIIIa-receptor on NK-cells [18] and the FcγRIIIb-receptor on neutrophils [19]. However, in other studies, using different antibodies, these results could not be confirmed [20], indicating that it is too early to draw general conclusions about the influence of glycosylation of IgG on Fc receptor binding and effector functions. Hence, altered glycosylation as observed in this study may be expected to modulate IgG effector functions by e.g. changing the conformation of the Fc part of IgG, thereby modulating affinity to the Fcγ-receptors.

Overall, decreased levels of non-galactosylated glycopeptides from IgG containing a bisecting GlcNAc were found to be an early feature of familial longevity detectable at middle age. Moreover, previous findings on degalactosylation with increasing age could be corroborated, as well as the sex-specific properties of this change. To provide a biological basis for these observations, it would be interesting to study the mechanisms by which protein glycosylation in B-cells is controlled. Further studies into the gene-expression of specific glycosylation-related enzymes in B-cells could also shine light on the biological processes underlying this association between IgG glycosylation and longevity.

## Methods

### Participants

In the Leiden Longevity study, Caucasian families were recruited if at least two long-lived siblings were alive and fulfilled the age-criterion of 89 years or older for males and 91 year or older for females, representing less than 0.1% of the Dutch population in 2001. In total, 956 long-lived proband siblings were included with a mean age of 94 (89–104), 1750 offspring with a mean age of 61 (39–81) and 758 partners with a mean age of 60 (36–79) [12].

The study protocol was approved by the Leiden University Medical Centre Ethical Committee and an informed consent was signed by all participants prior to participation in the study.

### IgG purification and glycosylation analysis

From a subset of the Leiden Longevity Study, consisting of 1287 offspring and 680 partners, immunoglobulin G was purified from citrate plasma samples in 22 96-wells filter plates using a Protein A affinity purification step as previously published [21]. In short, 2 µl of plasma was added to 15 µl Protein A coated beads in 185 µl PBS in a 96-well plate, and incubated at room temperature for 1 h. After washing, IgGs were eluted using 100 mM formic acid. After tryptic digestion of the isolated IgGs, peptides were purified using a C_18_-SPE plate.

Large scale analysis of IgG glycosylation profiles was performed using MALDI-TOF-MS. Samples were spotted with α-cyano-4-hydroxy-cinnamic acid matrix (5 mg/ml in 50% acetonitrile) and IgG glycosylation patterns were recorded on an Ultraflex II MALDI-TOF/TOF mass spectrometer (Bruker Daltonics, Bremen, Germany) which was operated in reflectron positive mode. N = 100 shots were acquired per position, and spectra were acquired from n = 20 different positions per spot, resulting in a sum spectrum obtained by accumulation from 2000 spectra per sample spot. Calibration was performed on a peptide calibration standard. The data was baseline subtracted and the intensities of an identical, defined set of 12 glycopeptide peaks (6 glycoforms for IgG1 and 6 for IgG2, see Figure 1) were automatically defined for each spectrum. For all glycoforms, relative intensities were calculated to the signal intensities of the H_4_N_4_F_1_ (**B**) glycopeptides which were set at 100. To evaluate the effect of bisecting GlcNAc, the ratios between H_3_N_5_F_1_ and H_3_N_4_F_1_ (**D**/**A**) and H_5_N_4_F_1_ and H_5_N_5_F_1_ (**F**/**C**) were calculated.

### Statistical analyses

To obtain normally distributed variables for IgG glycosylation, a log transformation was performed on all IgG variables. The samples of 1967 participants were divided over 22 individual plates to record IgG glycosylation patterns. Overall batch effects were assessed by Principal Component Analysis (PCA). Over 79% of the total variance was explained by the first two principal components (PCs). Visual inspection of box plots of the individual PC scores did not indicate heterogeneous within-plates variance, but mean (or median) values of some plates deviated from other plates. Therefore, location (mean) batch adjustment was employed to correct for batch effects [22].

To obtain valid standard errors which take into account familial correlations, empirical standard errors were calculated. P-values <0.05 were regarded statistically significant. First, linear regression was used to explore relationships between each of the response variables, IgG glycosylation values, and covariates - age and sex - adjusting for the family status. Next, the inclusion of an interaction term between sex and age in the linear regression model produced highly significant results. Further stratification according to offspring -partner and to young-old showed that the relationship between age and glycoforms differs for the sex and offspring-partner combinations and for the sex and young-old combinations, indicating the presence of three way interactions. Therefore, we chose a cut-off age of 60 years -which was the mean age- to create two separate data sets.

To determine potential biomarkers for longevity, logistic regression was applied to investigate whether IgG glycosylation value (independent variable) was predictive in classifying the family status after adjustment for age, sex, and their interaction. In that respect, the response variable (the family status) is coded as 0 ( =  partner) and 1 ( =  offspring of long-lived sibling). Based on the interaction study (Figure 3), stratified analyses were conducted using logistic regression for the family status - adjusting for sex, age and their interaction: one with individuals younger than 60 (n = 1073) and the second with individuals older than 60 years of age (n = 894).

To generate ROC curves, only subjects younger than 60 years of age were included. First, the effects of age were estimated using only the partners (as they are not enriched for longevity); the values for IgG1 **D**, IgG1 **E** and IgG2 **D** were age-adjusted using these parameters. By fitting the logistic model with binary outcome (offspring  = 0, partners  = 1), and each of the three IgG glycoforms together with sex as covariates, ROC curves were obtained.

Analyses were performed using STATA 10 (StataCorp LP, College Station, Texas, USA) and R version 2.9.0 (R Development Core Team).
